# Knowledge and barriers to pain management among cancer caregivers in Malaysia: a cross-sectional study

**DOI:** 10.7717/peerj.21281

**Published:** 2026-05-14

**Authors:** Syaza Syahirah Shamsuddin, Lili Husniati Yaacob, Siti Suhaila Mohd Yusoff, Fahisham Taib

**Affiliations:** 1 Department of Family Medicine, Universiti Sains Malaysia, Kubang Kerian, Kelantan, Malaysia; 2 Department of Paediatrics, Universiti Sains Malaysia, Kubang Kerian, Kelantan, Malaysia

**Keywords:** Knowledge, Barriers, Pain management, Cancer caregivers

## Abstract

**Introduction:**

Pain control among cancer patients remains inadequate despite the availability of effective pharmacological treatments. Caregivers’ knowledge of pain management is crucial to overall pain management. The objective of the study is to determine the level of knowledge on pain management among family caregivers of patients with cancers and explore the associated factors related to barriers in pain management.

**Materials and Method:**

A cross-sectional study involving family caregivers of patients with cancer attending Oncology Clinic was conducted from December 2022 until June 2023. The study tools included: (1) sociodemographic data of the family caregiver and clinical characteristics of cancer patients (2) the Malay version of the Family Pain Questionnaire (FPQ) which assesses the caregiver’s knowledge of pain management (3) the Malay version of Barries Questionnaire-13 which assess barriers toward pain management, and (4) the Malay version of Brief Pain Inventory (BPI) which assess patients’ pain experience. The data was analyzed using simple and multiple linear regression.

**Results:**

A total of 174 caregivers were recruited for this study. The mean knowledge score among caregivers was 47.5 ± 10.39. The mean score for BQ-13 was 29.14 ± 9.98. Multiple linear regression analysis showed that monthly income (*r* = 0.001 (95% CI [0.00–0.002]), *p* = 0.024), single status (*r* = 4.04 (95% CI [1.25–6.84]), *p* = 0.005), caregiver knowledge (*r* = 0.31 (95% CI [0.18–0.44]), *p* ≤ 0.001), and degree of pain experience among cancer patients (*r* = 0.08 (95% CI [0.04–0.13]), *p* ≤ 0.001) are the associated factors for barriers toward cancer pain management.

**Conclusion:**

The knowledge of pain management among family caregivers in our study was comparable to other previous studies. Level of knowledge, single status, pain experience, and monthly income had significant associations with barriers to pain management. The findings highlight the need for targeted educational support to address knowledge gaps and reduce barriers to effective pain management.

## Introduction

Pain is one of the most common symptoms of cancer, and it has a substantial negative influence on the quality of life and general well-being of patients. Its prevalence is on the rise throughout and beyond cancer treatment, with an overall prevalence of 44.5% (Snijders et al., 2023; Mestdagh, Steyaert & Lavand’homme, 2023). The WHO “analgesic ladder” has been the cornerstone of cancer pain treatment since 1986, and powerful opioids are the recommended course of treatment for patients with advanced cancer. The use of morphine and other potent opioids holds significance in treating cancer pain (Bennett, Paice & Wallace, 2017). Nonetheless, up to 50% of palliative care patients getting intensive opioid treatment appear to have an opioid phobia, with their top concerns being drug addiction, side effects, and dying, which continues to be one of the major obstacles to effective cancer pain management (Graczyk, Borkowska & Krajnik, 2019).

Family caregivers play a pivotal role in managing cancer pain. As cancer treatment is increasingly being provided in outpatient settings, managing pain is one of the most difficult aspects of a caregiver’s work throughout cancer treatment (Ferrell, 2019). Pain management has been recognized as a challenge since it calls on family caregivers to overcome their fears and concerns about pain management and requires them to have knowledge and abilities in pain assessment and management (Chi et al., 2023). To effectively manage pain, caregivers must be knowledgeable about cancer pain management as they are needed in managing drug side effects, monitoring pain, and administering care, however, they typically are untrained or unsupported when providing care (Rafii, Taleghani & Khatooni, 2020). Few studies showed that family caregivers’ lack of expertise in identifying pain and administering pain management contributes to inadequate pain control, which in turn results in undertreatment of cancer pain (Nayak, George & Vidyasagar, 2018; McPherson et al., 2014). Aside from that, it was discovered that inadequate pain management may be caused by the anxieties, misperceptions, and attitudes of caregivers regarding pain medications and cancer pain itself (Rafii, Taleghani & Khatooni, 2020). It is important to increase caregivers’ understanding of managing cancer pain as it was observed that caregivers with higher pain management expertise had significantly fewer barriers to cancer pain management (Vallerand et al., 2007).

Fear of opioid addiction, fear of drug intolerance, fear of injections, fear of analgesic side effects, the idea that “good” patients do not complain about pain, and the notion that pain indicates the progression of the disease are common attitudes among both cancer patients and their caregivers that contribute to the undertreatment of cancer pain (Lou & Shang, 2017). Caregivers and patients often have similar views towards the usage of opioids in controlling pain and caregivers frequently express misconceptions and worries about opiate addiction and analgesic side effects as obstacles to attaining adequate pain management (Konstantis & Exiara, 2018). In Malaysia, cancer pain management faces unique challenges. Community-based palliative care services remain limited, particularly in rural areas and structural gaps in service delivery have been described (Kher et al., 2021). Access to opioids such as morphine is tightly regulated, and misconceptions regarding addiction and side effects are common among patients and caregivers (Yc et al., 2013; Mohd Azmi et al., 2022). These structural and cultural factors may contribute to suboptimal pain control. Therefore, evaluating caregiver knowledge and perceived barriers within the Malaysian context is crucial for informing targeted educational interventions.

## Materials & Methods

This cross-sectional study was conducted at Oncology Clinic Hospital Universiti Sains Malaysia, from the 1st of December 2022 until the 1st of June 2023 involving primary caregivers of patients with cancer, who accompanied the patients coming for follow-up at Oncology Clinic Hospital Universiti Sains Malaysia (Hospital USM). The study’s criteria were primary caregivers of patients with cancer who are more than 18 years old and able to understand the Malay language. The exclusion criteria were caregivers who have mental impairment that is associated with a loss of sense of reality such as schizophrenia, bipolar disorder, acute psychosis, or dementia, and caregivers who are paid or professional caregivers. This study was approved by the Human Research Ethics Committee, Universiti Sains Malaysia (USM/JEPeM/22040274) and the Medical Research & Ethics Committee (NMRR ID-22-01521-KKU (IIR). The sample size of the study was calculated by using Power and Sample Size Calculation (PS) software version 3.1.2. The required sample size was calculated with the power of study set at 0.80 the α value was defined as 0.05 and the detectable difference (δ) was 0.34 which was calculated from the mean BQ scores of the female sex (Lin, 2000). The analysis revealed minimal sample size of the study was 176, and after considering 10% of non-responders into account, the estimated sample size was 194.

### Study tools

The first part of the questionnaire includes caregivers’ sociodemographic data such as age, sex, ethnicity, marital status, education level, employment status, income status, relationship with patient and welfare financial support, and clinical characteristics of cancer patients such as age, sex, types of cancer, duration of cancer, stage of cancer, treatment modalities, type of pain medications and route of pain medications. The information on the clinical characteristics of the patients was obtained through medical records.

### Malay version of the Family Pain Questionnaire

The second part of the questionnaire consists of the Malay version of the Family Pain Questionnaire (FPQ). The FPQ is a questionnaire used to evaluate knowledge and experience of pain among family caregivers. It consists of two separate components, knowledge of family caregivers in managing cancer pain (nine items), and caregiver’s experience with pain (seven items) (Ferrell, Rhiner & Rivera, 1993). Only the knowledge section of the questionnaire was used in this study. A pilot study was carried out to perform pre validation of the FPQ knowledge component. It was translated into Bahasa Malaysia using forward and backward translation and was evaluated in a pilot study involving 60 caregivers of patients in the Oncology Clinic in Hospital Pulau Pinang. The internal consistency of the knowledge component is satisfactory with a coefficient alpha (Cronbach’s alpha) of 0.66. Scoring for the knowledge section is done by using a rating scale where the responses of each item range from 0 (agree) to 10 (disagree) with a maximum score of 90, and the mean score of the knowledge section was calculated for analysis. A higher score denotes less proficiency with pain management. The pilot study has the same ethical approval as the main study.

### Malay version of Barrier Questionnaire-13

The third part is the Barrier Questionnaire. The original Barrier Questionnaire (BQ) is a 27-item self-reported instrument used to measure patient-related barriers toward cancer pain management, developed by Sandra E. Ward in 1993 (Gunnarsdottir et al., 2002). BQ-13, a shorter version was developed in 2010, in which the questionnaire was reduced to 13 items, which consist of two domains; pain management domains and side effects domains (Gunnarsdottir, Serlin & Ward, 2005). The pain management domain includes seven items on fatalistic beliefs or misconceptions about cancer pain and its treatment, and the side effects domain consists of six items addressing barriers related to side effects of pain treatment. The BQ-13 is reliable as it demonstrated internal consistency as a total scale (*α* = 0.73) and stability *via* 4-week test-retest reliability (Boyd-Seale et al., 2010). This questionnaire has been used among caregivers with good reliability; Cronbach alpha range from 0.78 to 0.82 (Wilkie et al., 2017; Bonds Johnson et al., 2022). Scoring for BQ-13 is done by using a rating scale where the responses of each item range from 0 (do not agree at all) to 5 (agree very much), with a maximum score of 65. The total BQ-13 score and mean of total scores were calculated and used for analysis. Higher scores indicate a higher level of barriers to cancer pain treatment. The BQ-13 also underwent pre validation in the same pilot study described above. It was translated into Bahasa Malaysia using forward and backward translation The internal consistency of the Malay version was satisfactory with a coefficient alpha (Cronbach’s alpha) of 0.69. Family Pain Questionnaire (FPQ) and Barrier Questionnaire (BQ-13) were answered by caregivers in this study.

### Malay version of Brief Pain Inventory

The final instrument is the Brief Pain Inventory (BPI). The Brief Pain Inventory measures a patient’s pain experience by rating the severity of pain and the degree to which pain interferes with a patient’s daily function (Cleeland, 2009). The pain intensity domain consists of four items: worst pain in the last 24 h, least pain in the last 24 h, average pain experience, and current pain experience. Each item in the pain intensity is rated on a Likert scale of 0 to 10.; with 0 as the least pain and 10 as the worst pain. The pain intensity score is the mean score of the four items. A total score of 1–3 is categorized as mild pain, 4–7 as moderate pain, and 8–10 as severe pain. A higher score indicates more severe pain.

The pain interference domain consists of seven items assessing pain interference in different aspects of a patient’s life such as general activity, mood, walking, work, enjoyment of life, relationship with others, and sleep. Pain interference is also presented as a numeric rating scale, with 0 as no interference, and 10 as complete interference. The pain interference score is the mean score of the seven items. A higher score indicates greater interference with daily activities.

Pain experienced by cancer patients was calculated using the total score of pain intensity and pain interference. The usage of the BPI-total score has been proven reliable in assessing pain experience among patients attending in Emergency Department (Im et al., 2020). The Cronbach’s alpha reliability of BPI ranges from 0.77 to 0.91 (Cleeland & Ryan, 1994). The Malay version of BPI was translated and validated in 2005 with reliability ranging from 0.61 to 0.88 (Aisyaturridha, Naing & Nizar, 2006). The BPI questionnaire section was given to patients to answer instead of caregivers to assess the patient’s pain experience.

This study utilized the convenience sampling method where eligible participants were identified from the patient registry at the oncology clinic. The participants were identified during their visit to the clinic and approached individually. Written informed consents were obtained from both caregivers and patients, and the questionnaires were given to be completed. The questionnaire completion took between 15–20 min. The caregivers were required to answer all three parts of the questionnaire (sociodemographic and clinical data, Malay version of FPQ, and Malay version of BQ-13). Meanwhile, patients were required to answer the Malay version of BPI. Patient medical records from the clinic were reviewed after the completion of the questionnaires for information regarding the clinical characteristics of the patients.

### Data analysis

The data were analyzed using SPSS version 24 (IBM Corp., Armonk, NY, USA). Descriptive statistics and frequencies were generated for sociodemographic and clinical characteristics of caregivers and patients. Descriptive analyses were also used to determine the level of knowledge and barriers regarding cancer pain among caregivers and were reported in the form of mean and standard deviation. Variables were first examined using simple linear regression. Variables demonstrating theoretical relevance or statistical significance set at *p* < 0.25 in univariate analysis were subsequently entered into the multiple linear regression model using the forward method. This approach was selected to ensure inclusion of clinically relevant predictors. Model assumptions, multicollinearity, and interaction effects were assessed and no violations were detected.

## Results

A total of 194 eligible caregivers were approached. Ten declined consents, and ten completed the questionnaires incompletely, resulting in 174 valid responses (response rate: 89.6%).

### Caregiver and patient characteristics

The mean age of caregivers was 37.68 ± 14.36 years. Most of them were female, 53.4% (*n* = 93), of Malay ethnicity 96.6% (*n* = 168), and were married 58% (*n* = 101). Monthly household income among caregivers was less than RM1600, and most of them were from the B40 income group 94.8% (*n* = 165) (Table 1). The average age of our patients was 54.25 years, with female patients making up 66.1% (*n* = 115) of them, and breast cancer (37.9%, *n* = 66) was the commonest diagnosis. The most common caregiver-patient relationships were children (47.1%) and spouses (29.3%). Most of the patients were in stage 3 or 4 (73%; *n* = 127) and receiving chemotherapy and/or radiotherapy as treatment (68.4%;119). Most of the cancer patients received non-opioid medications (79.9%; *n* = 139) for pain relief (Table 2).

**Table 1:** Sociodemographic data of caregivers (*n* = 174).

Sociodemographic characteristics	Mean (SD)	n (%)
Age (years)	37.68 (14.36)	
**Gender**		
Male		81 (46.6)
Female		93 (53.4)
**Race**		
Malay		168 (96.6)
Non-Malay		6 (3.4)
**Marital status**		
Married		101 (58.0)
Single		73 (42.0)
**Employment status**		
Employed		76 (43.7)
Un-employed		98 (56.3)
Monthly household income (RM)	1,374 (1,600)	
**Income Group**		
B40 (<RM4849)		165 (94.8)
M40 (RM 4850- RM 10959)		9 (5.2)
**Education level**		
No schooling/Primary/Secondary		7 (4.0)
Tertiary		167(96.0)
**Relationship with patient**		
Spouse		51 (29.3)
Parents		8 (4.6)
Children		82 (47.1)
Others		33 (19.0)
**Financial Aid**		
Yes		20 (11.5)
No		154 (88.5)
**Duration of caretaker**		
1–12 months		112 (64.4)
13–24 months		28 (16.1)
>25 months		34 (19.5)
**Living with patient**		
Yes		143 (82.2)
No		31 (17.8)

**Table 2:** Clinical data of cancer patients (*n* = 174).

Variables		n (%)
Diagnosis		
Breast		66 (37.9)
Colon		32 (18.4)
Nasopharyngeal		16 (9.2)
Lung		9 (5.2)
Prostate		8 (4.6)
Head & neck		8 (4.6)
Sarcoma		6 (3.4)
Lymphoma		5 (2.9)
Brain		5 (2.9)
Cervical		5 (2.9)
Uterine		3 (1.7)
Others		9 (1.1)
Duration of Cancer		
1–12 months		107 (61.5)
13–24 months		31 (17.8)
>25 months		36 (20.7)
Stage of Cancer		
Stage 1 or 2		47 (27.0)
Stage 3 or 4		127 (73.0)
Type of treatment		
Chemotherapy and/or radiotherapy		119 (68.4)
Chemotherapy, radiotherapy, and surgery		45 (25.9)
Surgery and Others		10 (5.7)
Type of Pain Medications		
Opioids		35 (20.1)
Non-opioids		139 (79.9)
Route of Medications		
Oral		172 (98.9)
Others		2 (1.1)

The overall total pain severity mean score was 7.16 (9.89) which indicated a moderate level of pain severity endured by our patients. Most patients (56.3%) did not report any pain over the 24 h, while 10.3% reported having mild pain, 20.1% reported having moderate pain and 13.2% reported experiencing severe pain in the last 24 h. Our patients have described a minimal level of pain interference with a mean score of 19.3 (23.05) out of a maximum score of 70. Most of the patients (58.6%) reported no interference, while only 12.1% had severe interference in daily activities. The overall pain experience score of patients in the study (which combines both pain intensity and interference) was mild with a score of 26.11 (29.68) out of a maximum score of 110 (Table 3).

**Table 3:** Patient pain experience.

Items	Mean (SD)
Worst pain in last 24 h	2.43 (3.29)
Least pain in last 24 h	1.37 (2.29)
Average pain experience	1.82 (2.63)
Current pain experience	1.56 (2.43)
Total (Pain interference)	19.3 (23.05)
General activity	2.92 (3.55)
Mood	2.99 (3.59)
Walking	2.56 (3.44)
Normal work	2.89 (3.69)
Relationship with others	2.13 (3.12)
Sleeping	2.82 (3.67)
Enjoyment of life	2.99 (3.64)

### Knowledge and barrier score

The mean knowledge score among caregivers was 47.5 ± 10.39 out of a maximum score of 90. The three items with the highest score indicating lowest knowledge were regarding tolerance (7.08 ± 2.62), disease progression (6.94 ± 2.59), and physiological dependence due to analgesics use (6.59 ± 2.98). Meanwhile, the lowest score was in the item “Most cancer patients on pain medicines will become addicted to the medicines over time” with a mean score of 3.24 ± 2.98 (Table 4).

**Table 4:** Knowledge score among caregivers.

Items of knowledge questionnaire	Mean (SD)
Cancer pain can be effectively relieved.	3.62 (2.62)
Pain medicines should be given only when pain is severe.	6.59 (2.98)
Most cancer patients on pain medicines will become addicted to the medicines over time.	3.24 (2.96)
It is important to give the lowest amount of medicine possible to save larger doses for later when the pain is worse.	7.08 (2.62)
It is better to give pain medications around the clock (on a schedule) rather than only when needed.	4.94 (3.42)
Treatments other than medications (such as massage, heat, relaxation) can be effective for relieving pain.	5.06 (2.79)
Pain medicines can be dangerous and can often interfere with breathing.	4.50 (3.08)
Patients are often given too much pain medicine.	3.72 (2.93)
If pain is worse, the cancer must be getting worse.	6.94 (2.59)

The mean score for BQ-13 was 29.14 ± 9.98. The mean score of barriers related to pain management was 2.75 ± 0.64. The highest item scored in the pain management domains was misconception regarding reporting pain would “distract the physician” Meanwhile, the lowest score in pain management domains was misconceptions regarding the “harmful effects” of pain medications. The highest score regarding barriers related to side effects was on drowsiness (Table 5).

**Table 5:** Caregivers’ barriers towards pain management.

Subscales	Mean (SD)
BQ-13 scores	29.14 (9.98)
Domains	
**Pain Management Domains**	2.75 (0.64)
Disease Progression	3.24 (1.35)
Fear of injections	2.18 (1.67)
Fatalism	1.87 (1.45)
Harmful effects	1.79 (1.56)
Be good	2.10 (1.80)
Distracting physician	3.37 (1.46)
Physiological effects	2.92 (1.44)
Easier to have pain than side effects	2.14 (1.44)
**Side Effects Domains**	
Drowsiness	2.30 (1.68)
Confusion	2.13 (1.63)
Nausea	2.26 (1.72)
Doing embarrassing things	1.09 (1.20)
Constipation	1.87 (1.69)

### Associated factors with barriers

Simple linear regression was used to examine the associated factors for barriers to cancer pain management among caregivers. Multiple linear regression showed that knowledge of caregivers, marital status of the caregivers, monthly household income of caregivers, and degree of pain experience among the cancer patients were significant associated factors with barriers to cancer pain management among caregivers (*p* < 0.05) (Table 6). Single caregivers showed an increase of 4.04 units in barriers score as compared to married caregivers. Caregivers, who had higher monthly household incomes, showed an increase of 0.001 units in barrier score compared to those with higher household incomes. 1 point increase in knowledge score, whereby the higher score means poorer knowledge, contributed to an increase in 0.31-point barriers score to cancer pain management. Furthermore, a one-point increase in total score for pain experience among cancer patients was associated with an increase in 0.08 points of barrier score to cancer pain management. With the four significant variables, the model explained 20.6% of the variation in the barrier scores in the study sample (R^2^ = 0.206).

**Table 6:** Associated factors for barriers to cancer pain management (*n* = 174).

Variables	SLR^a^			MLR^b^		
	B^c^ (95% CI^d^)	t stat	*p* value	Adj. B^e^ (95% CI^d^)	t stat	*p* value
***Sociodemographic Caregiver***						
Caregiver’s Age (years)	−0.04 (−0.14, 0.07)	−0.66	0.509			
Caregiver’s Gender				
Male						
Female	−1.31 (−4.30, 1.68)	−0.87	0.388			
Caregiver’s Race						
Malay						
Non-Malay	0.73 (−4.63, 6.08)	0.27	0.789			
Caregiver’s Marital status						
Married						
Single	3.04 (0.04, 6.04)	2.00	0.047	4.04 (1.25, 6.84)	2.85	0.005
Caregiver’s Employment Status						
Employed						
Unemployed	−1.34 (−4.36, 1.67)	−0.88	0.380			
Caregiver’s Monthly Household Income	0.001 (0.00, 0.001)	1.19	<0.001	0.001 (0.00,0.001)	2.27	0.024
Caregiver’s Household Income Group						
B40 (<RM4849)						
M40 (RM 4850- RM 10959)	3.84 (−2.90, 10.58)	1.12	0.262			
Caregiver’s Education Level						
No schooling/Primary/Secondary						
Tertiary	0.59 (−7.03, 8.21)	0.15	0.879			
Relationship Caregivers with Patient						
Spouse						
Parents	−0.25 (−7.81, 7.30)	−0.07	0.947			
Children	0.87 (−2.67, 4.41)	0.49	0.627			
Others	0.59 (−3.85, 5.02)	0.26	0.795			
Financial Aid						
Yes						
No	2.98 (−1.70, 7.66)	1.256	0.210			
***Clinical Factors of Caregivers***				
Duration of Caretaker				
1–12 months						
13–24 months	−0.63 (−4.82, 3.55)	−0.30	0.765			
>25 months	−0.92 (−4.80, 2.96)	−0.47	0.640			
Caregiver’s living with Patient						
Yes						
No	0.85 (−3.06, 4.77)	0.43	0.668			
Knowledge of Pain in Caregiver’s (FPQ score)	0.33 (0.20, 0.47)	4.86	<0.001	0.31 (0.18, 0.44)	4.63	<0.001
***Clinical factors of patients***						
Patient’s Age (years)	−0.01 (−0.11, 0.01)	−0.16	0.875			
Gender						
Male						
Female	−0.13 (−3.29, 3.04)	−0.08	0.938			
Duration of Cancer						
1–12 months						
13–24 months	−0.87 (−4.91, 3.16)	−0.43	0.670			
>25 months	−1.25 (−5.06, 2.57)	−0.65	0.520			
Stage of Cancer						
Stage 1 or 2						
Stage 3 or 4	0.25 (−3.13, 3.62)	0.15	0.885			
Type of Treatment						
Chemotherapy and/or radiotherapy						
Surgery and Others	1.98 (−4.54, 8.49)	0.60	0.550			
Chemotherapy, radiotherapy, and surgery	0.78 (−2.68, 4.24)	0.44	0.659			
Type of Pain Medications						
Opioids						
Non-opioids	0.32 (−3.42, 4.05)	0.17	0.868			
Route of Medications						
Oral						
Others	−5.20 (−19.23, 8.83)	−0.73	0.466			
Pain Experience	0.09 (0.05, 0.14)	3.80	<0.001	0.08 (0.04, 0.13)	3.59	<0.001

**Notes.**

a Simple Linear Regression.

b Multiple Linear Regression.

c Crude regression coefficient.

d Confidence Interval.

e Adjusted regression coefficient.

The forward multiple linear regression method was applied. Model assumptions are fulfilled.

There were no interactions among independent variables. No multicollinearity was detected (VIF less than 10). The assumption was checked, and there was no violation.

Coefficients of determination (R^2^) = 0.206.

## Discussion

Knowledge of caregivers regarding cancer pain is important for them to help control pain among patients with cancer. This study showed that the total knowledge score among our respondents was 47.5. Lou & Shang (2017) showed a moderate level of pain knowledge among caregivers of cancer in Beijing with a total score of 43.69. Similar findings using a similar instrument, the Family Pain Questionnaire (FPQ) were found among caregivers in Germany which scored 41.7 indicating a moderate level of cancer pain knowledge (Kizza & Maritz, 2019). While comparable to international data, this score suggests suboptimal knowledge, which may hinder caregivers’ ability to manage pain effectively Our caregivers had the lowest knowledge on items related to the development of dependence and tolerance towards pain medications and the use of pain medication as the disease progressed which is similar to the finding in a study conducted in China (Ma et al., 2022). Caregivers must have correct knowledge of pain management as patients’ attitudes towards cancer pain were influenced by their caregiver’s knowledge (Makhlouf et al., 2019).

The barrier to cancer pain management among caregivers in our study was comparable to previous studies (Bonds Johnson et al., 2022; Wilkie et al., 2017). One area that was a main concern for our caregivers was the communication aspect, especially when disclosing discomfort to physicians. They were also concerned with the physiological effects of opioids with negative views on the outcome. These findings are similar to a finding in a study conducted in Georgia which found that the three subscales with the greatest scores were fear of opioid addiction, diverting the doctor’s attention, and worry about the opioid-related side effects of constipation (Bonds Johnson et al., 2022). Meanwhile, Wilkie et al. (2017) found that the three highest concerns were seen in areas of side effects of opioids such as “addiction”, “pain medication causing confusion” and “pain medication causing one to do embarrassing things”. Saifan et al. (2015) concluded that total mean scores of the BQ-II Arab version for Jordanian patients and their families were high with four major factors identified in impeding effective pain relief among patients and caregivers such as the use of opioids, side effects of opioids, communication, and cultural beliefs. A study by Ferrell (2019) revealed caregivers’ worries about the administration of medications, their ignorance of pharmaceutical options, how to address breakthrough pain, and how to differentiate pain from other symptoms. Furthermore, several studies showed that a lack of knowledge and misconception regarding opioids influence caregivers in ensuring adequate pain management among their loved family members with cancer (Lou & Shang, 2017; Makhlouf et al., 2019; Kizza & Maritz, 2019).

This study showed there is an association between caregivers’ level of knowledge and their barriers to cancer pain management. Our study showed that poorer knowledge about cancer pain is associated with higher barriers to effective cancer pain management. These findings are consistent with Vallerand et al. (2007) who also showed caregivers with better knowledge have significantly fewer barriers to cancer pain management. Many studies conducted before showed that poor caregivers’ knowledge influences their attitudes toward cancer management thus making it difficult to achieve good pain control (Lou & Shang, 2017; Makhlouf et al., 2020; Ma et al., 2021). Home-based education intervention towards family caregivers has shown to be effective in improving knowledge and thus resulting in self-efficacy for pain management at home (Kizza & Muliira, 2019). A recent systematic review and meta-analysis by Schoth et al. (2025) further supports the effectiveness of structured home-based symptom management programmes for family caregivers, showing improvements in caregiver competence and patient symptom outcomes across physical domains. These findings reinforce the importance of implementing structured educational and support strategies for caregivers within the Malaysian healthcare context, particularly where outpatient and home-based cancer care is common. This indicates the need for education delivered to family caregivers about how to manage pain to prepare them at home. While institutional and hospice-based palliative care teams play a vital role in cancer pain management, this study focuses on the home care setting where caregivers serve as frontline providers. In many parts of Malaysia, particularly rural regions, access to hospice or fully staffed palliative teams remains limited (Ministry of Health Malaysia, 2010). Therefore, equipping family caregivers with adequate knowledge is essential to ensuring effective pain management in the home environment. Barriers to pain management were also influenced by the sociodemographic background of caregivers and this was corroborated by research in 2020, which found that sociodemographic characteristics were among the common barriers to cancer pain treatment (Abdullah, Khraim & Al-Tawafsheh, 2020). Interestingly, our study found that single status was associated with an increase in barriers to pain management. Social support and relationship quality were found to buffer the negative effects of subjective caregiver burden on mental health (Tough, Brinkhof & Fekete, 2022). As primary caregivers, spouses frequently support their partner’s pain management requirements and offer helpful assistance. Previous study has shown that caregivers of individuals with cancer experienced decreased quality of life due to inadequate social support (Rostami et al., 2023). This might partly explain the association between single caregivers with barriers to pain management. However, there is no similar study that has shown how the single status of caregivers causes differences in knowledge, attitude, or practices toward cancer pain management. Thus, there is room for exploration in the future regarding how this sociodemographic determinant affects cancer pain management.

Previous studies have found that financial burden is another sociodemographic aspect that raises hurdles for caregivers (Vahidi et al., 2016; Gabriel, 2019; Kusi et al., 2020). Interestingly, our study found that higher income was associated with slightly higher barriers to pain management, contrary to the common expectation that financial stability would reduce obstacles. However, the effect size is very small (0.001 per unit increase in income), suggesting that while statistically significant, the practical impact might be minimal. One possible explanation is that higher-income caregivers may have greater expectations for pain management and access to treatments, leading to increased frustration and perceived barriers when those expectations are not met. Additionally, higher socioeconomic status may correlate with increased autonomy in decision-making, leading to greater skepticism or concerns about opioid use and pain management strategies recommended by healthcare professionals. Cultural factors may also play a role, as caregivers from wealthier backgrounds might feel a heightened sense of responsibility for ensuring optimal care, amplifying their perceived barriers. This finding is in contrast to a study in Nigeria found there was a significant relationship between caregiver burden and characteristics of caregivers such as monthly income, income status, and duration of caregiving (Gabriel, 2019). These indicate that there are multifactorial factors contributing to the association between low income and barriers to cancer pain management.

In addition, we discovered that the more pain cancer patients endure, the more obstacles’ caregivers face in managing their discomfort. A similar finding has not been shown by other previous studies. However, a study showed that caregivers’ perception of patient’s pain interference was significantly associated with beliefs regarding pain management and side effects of medications (Bonds Johnson et al., 2022). Another study comparing the barrier ratings of patients and caregivers discovered that most patients reported under medicating their pain, and the overall barrier scores of caregivers significantly predicted how much analgesia the patients took (Lin, 2000). In contrast, Wilkie et al. (2017) discovered that although mean barrier scores remained high among patients and caregivers, they were not substantially correlated with the intensity of the patient’s pain. Our study showed similar findings in which the mean barrier score of caregivers was high in patients who reported severe pain as compared to the barrier score of caregivers who reported least, average, and having current pain in 24 h, however, were not significant to their pain intensity. However, our study did not explore barriers from the cancer patients themselves, thus there is still room for exploration in the future to see pain management outcomes by comparing patients’ and caregivers’ barrier scores.

The study’s strength lies in its local approach, which involves employing translated and validated questionnaires to evaluate cancer patients’ suffering and caregivers’ knowledge and barriers. Additionally, the study provides clinically relevant insights into the barriers caregivers face in managing cancer pain, which can inform targeted interventions to improve patient care. Despite these strengths, this study has several limitations. The cross-sectional design limits the ability to establish causality between caregiver characteristics and barriers to pain management. Additionally, the study was conducted at a single oncology clinic, which may reduce the generalizability of the findings to other healthcare settings in Malaysia. Self-reported data were used, which introduces the possibility of a recall bias or social desirability bias, as participants may have reported what they believed was expected rather than their actual perceptions. Another limitation is the lack of an in-depth exploration of cultural factors that may influence caregiver perceptions of pain management, which could provide a more comprehensive understanding of the barriers faced. Future research should consider longitudinal studies and qualitative approaches to further explore these dynamics. It is acknowledged that cancer pain is only one aspect of holistic palliative care. Nutrition, emotional support, socialization, and spiritual wellbeing are also important (Ferrell et al., 2018). Future studies should explore caregiver knowledge and challenges in these broader domains to provide a more comprehensive picture.

## Conclusions

In conclusion, our study showed gaps in knowledge regarding cancer pain among caregivers and barriers to achieving good cancer pain management. This study also ascertained that caregivers’ knowledge, single marital status, and monthly income contribute to barriers to cancer pain management. Our findings highlight the significance of education and support for caregivers to enhance their understanding of pain management and overcome the barriers they face. Moving forward, future research should focus on analyzing the impact of educational interventions and support programs targeting caregivers.

##  Supplemental Information

10.7717/peerj.21281/supp-1 Supplemental Information 1 STROBE checklist

10.7717/peerj.21281/supp-2 Supplemental Information 2 Raw data

## Additional Information and Declarations

### Data Availability

The following information was supplied regarding data availability: The raw data is available in the Supplemental File.
